# A RETREG1/FAM134B isoform switch regulates reticulophagy during myogenesis

**DOI:** 10.1080/15548627.2025.2494803

**Published:** 2025-04-25

**Authors:** Viviana Buonomo, Michele Cillo, Paolo Grumati

**Affiliations:** a Telethon Institute of Genetics and Medicine (TIGEM), Pozzuoli, Italy; b Department of Clinical Medicine and Surgery, Federico II University, Naples, Italy

**Keywords:** Autophagy, FAM134B, myogenesis, reticulophagy, RETREG1

## Abstract

During skeletal muscle development, the sarcoplasmic reticulum forms through the homotypic fusion of ER membranes from individual myoblasts. This involves significant ER remodeling, characterized by an overhaul of its proteomic landscape and the activation of reticulophagy. We described how RETREG1/FAM134B is implicated in both shaping ER morphology and degrading ER membranes through reticulophagy. Following myoblast differentiation, the classic RETREG1/FAM134B1 undergoes lysosomal degradation and is progressively replaced by the shorter RETREG1/FAM134B2 isoform. RETREG1/FAM134B2 is a truncated variant of RETREG1/FAM134B1 maintaining an identical C-terminal region, including the functional LIR, but with a partial loss of its reticulon homology domain. The switch between these two isoforms plays a crucial role in ER morphology and muscle development. Re-expressing *Retreg1/Fam134b2* in *retreg1/fam134b*-knockout myoblasts is both necessary and sufficient to rescue the abnormal proteomic landscape and prevent ER dilation. Conversely, the re-expression of *Retreg1/Fam134b1* only partially rescues ER defects. We highlighted the role of RETREG1/FAM134B isoforms and reticulophagy in maintaining proper ER dynamics during myogenesis.

The endoplasmic reticulum (ER) has the intrinsic ability to remodel its shape and size in response to cellular stimuli that occur throughout an organism’s lifespan. ER morphology is tuned by resident proteins and external forces that drive membrane bending, tubulation and vesiculation. Moreover, the elaborate and dynamic structure of the ER is linked to its functional roles within the cell. Each cell type has specialized functions that require well-defined ER morphology and protein composition.

Reticulophagy, the selective autophagic degradation of the ER, plays a key role in ER membrane remodeling. This process requires fragmentation of the ER network into discrete portions for delivery to lysosomes. Reticulophagy receptors, like the RETREG family and RTN3, contribute to the membrane remodeling through their reticulon homology domain (RHD). Additionally, their LC3-interacting region (LIR) enables them to interact with the autophagy machinery, ensuring that the fragmentation and turnover of ER membranes are in lockstep.

During myogenesis the ER undergoes extensive structural and proteomic reorganization to form the sarcoplasmic reticulum (SR). Single ERs, from individual myoblasts, fuse to generate a unified SR within myotubes. The molecular mechanisms behind the shaping of the ER network throughout this process are still unclear. Therefore, we investigated the temporal dynamics of ER remodeling during myogenesis.

We compared the full proteomes of undifferentiated myoblasts and differentiated myotubes highlighting significant alterations in the proteomic landscape of the ER, along with a notable enrichment of proteins associated with the lysosomal pathway. Therefore, we investigated whether reticulophagy plays a role in myoblast differentiation. We monitored reticulophagy flux during myoblast differentiation over 12 days. We noticed that in proliferating myoblasts, reticulophagy flux was maintained at a low basal level. As cells began to fuse, reticulophagy flux rapidly increases, peaking at the height of cell fusion and returning to its initial basal level after myotubes maturation.

We identified RETREG1/FAM134B as a key driver of reticulophagy during myogenesis [1]. The canonical RETREG1/FAM134B is expressed only in myoblasts while it is absent in mature skeletal muscle where it is progressively degraded and replaced by its shorter isoform, RETREG1/FAM134B2. *Retreg1/Fam134b2* expression is transcriptionally regulated, starting at day 4 of differentiation. Its promoter resides within the third intron of the *Retreg1/Fam134b* gene and shares no homology with the *Retreg1/Fam134b1* promoter, suggesting that the two isoforms are differentially regulated, possibly in a tissue- and stress-specific manner. In our settings, MYF6 preferentially binds to the *Retreg1/Fam134b2* promoter, while MYF5 associates with the *Retreg1/Fam134b1* promoter.

RETREG1/FAM134B2 lacks the cytosolic N-terminal domain and the first hairpin structure of RETREG1/FAM134B1’s RHD. The remaining protein, including the second hairpin element, the two amphipathic helices, and the C terminus (including the LIR) remain identical maintaining RETREG1/FAM134B2’s ability to function as a reticulophagy receptor. Structural modeling suggested that RETREG1/FAM134B2 preferentially adopts a rigid V-shaped conformation, in contrast to the highly dynamic RETREG1/FAM134B1. However, RETREG1/FAM134B2 retains the intrinsic ability to induce ER membrane vesiculation, albeit at a slower rate. Notably, in myotubes, RETREG1/FAM134B2 expression does not alter ER network integrity, whereas the RETREG1/FAM134B1 continues to actively vesiculate ER membranes.

Both isoforms can oligomerize. In myotubes, RETREG1/FAM134B2 favors homodimer formation that exhibits few interacting partners but shows a high affinity for ubiquitin modifiers. In contrast, RETREG1/FAM134B1 dimers interact with a broader range of proteins. Moreover, in RETREG1/FAM134B1, ubiquitination and phosphorylation sites are primarily located within or near the first portion of the RHD and are essential for protein clustering. RETREG1/FAM134B2 lacks these PTM sites but contains alternative ubiquitination and phosphorylation sites mainly in its C-terminal region, suggesting that these modifications may regulate oligomerization and interactors’ specificity. Therefore, RETREG1/FAMB134B2 emerges as a functionally distinct regulator of ER dynamics during muscle differentiation.

This represents the first case where the reticulophagy is driven by a dynamic switch between two isoforms of RETREG1/FAM134B. The unique structural properties of RETREG1/FAM134B2’s partial RHD must be responsible for its specialized function. In myotubes, the ER network adopts a more well-defined architecture to form interconnected tubules, making the switch between the RETREG1/FAM134B isoforms critical for establishing this new ER morphology.

We generated retreg1 knockout C2C12 cells and reconstituted them with expression of either RETREG1/FAM134B1, RETREG1/FAM134B2, or both. The *retreg1* knockout cells exhibit an enlarged ER with persistently dilated membranes, even after differentiation into myotubes. Proteomic analysis revealed significant differences between wild-type and *retreg1* knockout myotubes, with the loss of Retreg1/Fam134b leading to impaired reticulophagy flux and the accumulation of ER proteins. Reconstitution of *retreg1* knockout cells with human *RETREG1/FAM134B2 (HsRETREG1/FAM134B2)* successfully restores ER morphology, proteomic landscape, and reticulophagy flux to wild-type levels. In contrast, an HsRETREG1/FAM134B2 LIR mutant fails to rescue either the proteome landscape or ER morphology, underscoring the importance of the autophagy machinery. Surprisingly, HsRETREG1/FAM134B1, either alone or in combination with HsRETREG1/FAM134B2, only partially rescues the phenotype. This is likely due to an over-correction effect: while HsRETREG1/FAM134B2 reestablishes a physiological level of ER proteins, HsRETREG1/FAM134B1 excessively reduces protein levels, resulting in an imbalance in the opposite direction. This finding helps to explain why RETREG1/FAM134B1 must be degraded during myogenesis, as its persistence disrupts proper ER remodeling, preventing the phenotypic rescue of retreg1 knockout myotubes. Additionally, RETREG1/FAM134B2 must be expressed only when myoblasts start fusing. Premature expression negatively affects myotube differentiation, further emphasizing the tightly regulated nature of this isoform switch.

We provided the first evidence for a biological role of reticulophagy during myogenesis and we characterized RETREG1/FAM134B2 as an ER morphogen, whose precisely timed expression is essential for skeletal muscle development (Figure 1). The myogenic process relies on the finely tuned regulation of RETREG1/FAM134B isoforms, ensuring proper ER remodeling and function.

**Figure 1.**
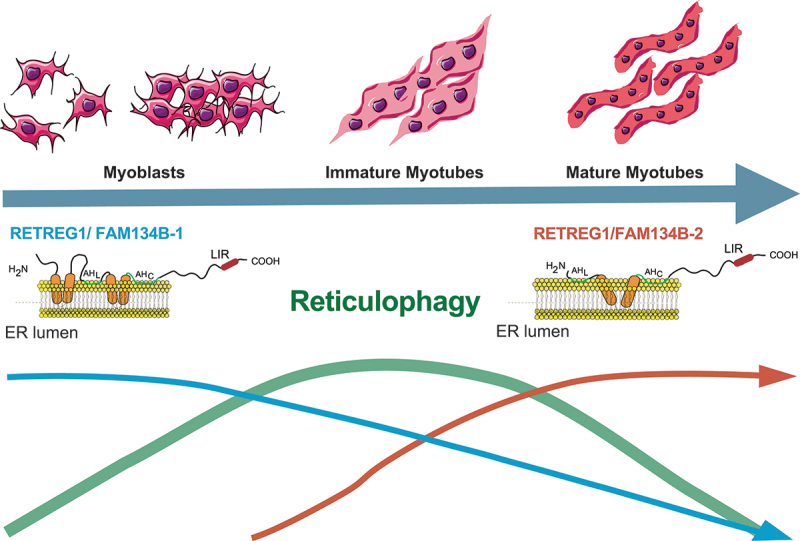
Temporal dynamics of reticulophagy and RETREG1/FAM134B isoform regulation during skeletal muscle differentiation. Myogenesis is illustrated from myoblasts, immature myotubes, and mature myotubes. The graph illustrates the transient nature of reticulophagy, which increases during differentiation, peaking around day 4 and then subsiding when myotubes mature. RETREG1/FAM134B1, the canonical isoform, is expressed in myoblasts but is progressively degrades as differentiation proceeds. In contrast, RETREG1/FAM134B2 expression is transcriptionally upregulated from day 4 onward, coinciding with increased reticulophagy activity. This isoform switch is crucial for ER remodeling and the formation of a structured sarcoplasmic reticulum in myotubes. AH: amphipathic helices.
